# Treatment with ascorbic acid and α-tocopherol modulates oxidative-stress markers in the spinal cord of rats with neuropathic pain

**DOI:** 10.1590/1414-431X20177097

**Published:** 2018-03-01

**Authors:** A.P.K. Riffel, M.C.Q. Santos, J.A. de Souza, T. Scheid, A. Horst, C. Kolberg, A. Belló-Klein, W.A. Partata

**Affiliations:** 1 Laboratório de Neurobiologia Comparada, Departamento de Fisiologia, Instituto de Ciências Básicas da Saúde, Universidade Federal do Rio Grande do Sul, Porto Alegre, RS, Brasil; 2 UNIVATES, Lajeado, RS, Brasil; 3 Centro Universitário da Serra Gaúcha, Caxias do Sul, RS, Brasil

**Keywords:** Total thiol, Superoxide anion generation, Nitric oxide, Lipid hydroperoxides, Hydrogen peroxide, Total antioxidant capacity

## Abstract

Vitamin E (vit. E) and vitamin C (vit. C) are antioxidants that inhibit nociception. The effect of these vitamins on oxidative-stress markers in the spinal cord of rats with chronic constriction injury (CCI) of the sciatic nerve is unknown. This study investigated the effect of intraperitoneal administration of vit. E (15 mg·kg^-1^·day^-1^) and vit. C (30 mg·kg^-1^·day^-1^), given alone or in combination, on spinal cord oxidative-stress markers in CCI rats. Adult male Wistar rats weighing 200–250 g were divided equally into the following groups: Naive (rats did not undergo surgical manipulation); Sham (rats in which all surgical procedures involved in CCI were used except the ligature), and CCI (rats in which four ligatures were tied loosely around the right common sciatic nerve), which received injections of vitamins or vehicle (saline containing 1% Tween 80) for 3 or 10 days (n=6/each group). The vitamins prevented the reduction in total thiol content and the increase in superoxide-anion generation that were found in vehicle-treated CCI rats. While nitric-oxide metabolites increased in vehicle-treated CCI rats 3 days after surgery, these metabolites did not show significant changes in vitamin-treated CCI rats. In all rats, total antioxidant capacity and hydrogen-peroxide levels did not change significantly. Lipid hydroperoxides increased 25% only in vehicle-treated CCI rats. These changes may contribute to vit. C- and vit. E-induced antinociception, because scavenging reactive oxygen species seems to help normalize the spinal cord oxidative status altered by pain.

## Introduction

Neuropathic pain, which arises as a direct consequence of a lesion or disease affecting the somatosensory system, affects 6–10% of the population and negatively impacts the quality of life (1). The pathophysiological mechanisms of neuropathic pain are not fully understood. The lack of effective analgesics has impelled a continuing search to find novel molecules that have beneficial effects in the management of neuropathic pain. Since reactive oxygen species (ROS), which include superoxide radicals, hydroxyl radicals, hydrogen peroxide (H_2_O_2_), nitric oxide (NO), and peroxynitrite, play an important role in neuropathic pain (2), antioxidant agents have been tested for its treatment (3,4).

Vitamin C (vit. C) and vitamin E (vit. E) are potent dietary antioxidants (5). Recently, we demonstrated that treatment with a combination of vit. C plus vit. E was more effective in treating chronic constriction injury (CCI)-induced neuropathic pain than these vitamins individually, which also showed an antinociceptive effect when given alone (4). In addition, these authors showed that co-administration of vit. C plus vit. E and gabapentin (an analgesic to treat neuropathic pain) induced a greater antinociceptive effect than gabapentin alone. Rats with CCI are one of the most commonly employed animal models of neuropathic pain, as CCI simulates the symptoms of chronic nerve compression that correspond to causalgia or complex regional pain syndrome in human patients (6).

According to Riffel et al. (4), the administration of vit. C plus vit. E induced changes in oxidative parameters in the injured sciatic nerve. In CCI rats that received the combination of vitamins, the total antioxidant capacity (TAC) increased (45%), while lipid hydroperoxide levels (a marker of pro-oxidant status) decreased (38%). However, that study did not assess oxidative parameters in the lumbosacral spinal cord, the region where most afferent fibers of the sciatic nerve enter. Evidence suggests that the main action site for ROS in neuropathic pain is the spinal cord (7). In addition, vit. E is a potent lipophilic chain-breaking antioxidant, found in biological membranes (8). Its most active isomer, α-tocopherol, is rapidly depleted in the body, requiring regeneration through other antioxidants present in the water-soluble portion of the cell, such as ascorbate (the monovalent anion of vit. C) (9). Ascorbate readily penetrates the central nervous system after oral administration (10). We postulated that the analgesic effect of vit. C and vit. E, alone or in combination, would involve modulation of ROS in the lumbosacral spinal cord. Therefore, our study assessed the effect of intraperitoneal (*ip*) administration of vit. C, vit. E, and vit. C plus vit. E (vits. C+E) on the total content of thiols and TAC, as markers of antioxidant status, in the lumbosacral spinal cord of rats with CCI. We also assessed the superoxide-anion generation (SAG) and the levels of lipid hydroperoxides, H_2_O_2_ and NO metabolites in this tissue, as markers of pro-oxidant status.

## Material and Methods

### Experimental animals and treatment

All animal procedures were approved by the Ethics Committee for Animal Experimentation of the Universidade Federal do Rio Grande do Sul (CEUA-UFRGS #23352). All efforts were made to minimize animal suffering and to reduce the number of animals used. Adult male Wistar rats, weighing 200–250 g, were randomly and blindly divided into three experimental groups (naive, sham and CCI), and each was further divided into four subgroups (n=12/subgroup), which received vit. C (30 mg·kg^-1^·day^-1^ L-Ascorbic Acid, Sigma Chemical Co., USA), vit. E (15 mg·kg^-1^·day^-1^ DL-alpha-tocopherol acetate, Sigma Chemical Co.), a combination of these vitamins in the same doses (vits. C+E) (4,11) or vehicle for 3 and 10 days (n=6/each treatment). The vitamins were freshly prepared in saline containing 1% Tween 80 (Merck, Germany) which was used as the vehicle. The administration started on the day of surgery (after recovery from anesthesia) and was performed daily at 5:00 pm by the same researcher (4).

### Induction of peripheral neuropathy by CCI

CCI was performed based on the procedure described by Bennett and Xie (12), with slight modifications according to Riffel et al. (4). After anesthesia (90 mg/kg ketamine and 10 mg/kg xylazine), the right common sciatic nerve was exposed via a mid-thigh incision. Proximal to the sciatic trifurcation, the nerve was freed of adhering tissue for about 7 mm, and four ligatures (4.0 chromic catgut, Shalon Fios Cirúrgicos Ltda., Brazil) were tied loosely around it, with a 1.0–1.5 mm interval between each ligature. After nerve ligation, the muscle and skin layer was immediately sutured with thread and a topical antibiotic applied. To expose the sciatic nerve in sham rats, all surgical procedures involved in CCI were used except the ligature.

### Mechanical threshold

Mechanical threshold was assessed by electronic von Frey apparatus (Insight, Brazil). A positive response was indicated by an abrupt withdrawal of the paw, and the intensity of the pressure was automatically recorded (in grams). A single trial consisted of five applications of the plastic tip, once every 5–10 s. The mean of five readings was taken as the threshold for a specific timing trial.

### Sample preparation

Rats were killed by decapitation and their lumbosacral spinal cord was promptly dissected out and divided transversely into three parts. The same portion always received the same preparation. Two parts were cooled in liquid nitrogen and processed to determine SAG and H_2_O_2_. A third part was homogenized in 1.15% KCl diluted 1:5 (w/v) containing 1 mM phenylmethylsulfonyl fluoride, centrifuged at 1000 *g* for 20 min at 4°C, and the supernatant was used for assays of total thiols, TAC, lipid hydroperoxides levels and NO metabolites.

### Determination of total thiol levels

Total thiol content was determined as described by Aksenov and Markesbery (13). Briefly, 30 μL of a sample was mixed with 1 mL of phosphate/EDTA buffer, pH 7.5, and 5,5′-ditiobis (2-nitrobenzoic) acid (DTNB, 10 mM). Control samples, which did not include DTNB, were run simultaneously. After 30 min of incubation at room temperature, the absorbance was read at 412 nm. Results are reported as mmol/mg tissue.

### Determination of TAC

TAC was determined with 2,2-azinobis-(3-ethylbenzothiazoline-6-sulfonic acid) radical cation, which in an acid medium is decolorized by antioxidants, according to their concentration and antioxidant capacity (14). Results are reported in µmol·eq trolox^-1^·g tissue^-1^.

### Estimation of superoxide anion generation (SAG)

Lumbosacral spinal cord SAG was estimated by using the reduced nitroblue tetrazolium (NBT) method of Wang et al. (15). Briefly, sections of fresh tissue from the lumbosacral spinal cord reacted with NBT to form formazan as an index of superoxide anion generation. The absorbance of formazan was determined spectrophotometrically at 540 nm.

The quantity of NBT reduction = A × V / (T × Wt × ε × l), where A is the absorbance of blue formazan at 540 nm, V is the volume of the solution, T is the time period (90 min) during which the rings were incubated with NBT, Wt is the blotted wet weight of the spinal cord portion, ε is the extinction coefficient of blue formazan (i.e., 0.72 L·mmol^-1^·mm^-1^), and l is the length of the light path. Results are reported as reduced NBT pmol·min^-1^·mg tissue^-1^.

### Determination of H_2_O_2_

The assay was based on horseradish peroxidase (HRPO)-mediated oxidation of phenol red by H_2_O_2_, leading to the formation of a compound that absorbs at 610 nm. Sections of fresh tissue from the lumbosacral spinal cord were incubated for 30 min at 37°C in 10 mM phosphate buffer (140 mM NaCl and 5 mM dextrose). The supernatants were transferred to tubes with 0.28 mM phenol red and 8.5 U/mL HRPO. After 5 min incubation, 1 mol/L NaOH was added, and the solution was read at 610 nm. The results are reported as μmol H_2_O_2_/g tissue (16).

### Determination of lipid hydroperoxides levels

Lipid hydroperoxides were measured by oxidation of Fe^2+^ by LOOH in an acid medium containing xylenol orange dye, which forms a complex with Fe^3+^, as described by Jiang et al. (17). Results are reported as µmol/g tissue.

### Determination of NO metabolites

To measure NO metabolites, nitrites (NO_2_) were determined using the Griess reagent, in which a chromophore with a strong absorbance at 540 nm is formed by reaction of NO_2_ with a mixture of 0.1% naphthylethylenediamine and 1% sulfanilamide. Nitrates (NO_3_) were determined as total NO_2_ (initial NO_2_ plus NO_2_ reduced from NO_3_) after their reduction using NO_3_ reductase from *Aspergillus* species in the presence of nicotinamide dinucleotide phosphate acid. A standard curve was established with a set of serial dilutions (10^−8^ to 10^−3^ mol/L) of sodium NO_2_. Absorbance at 540 nm was obtained (spectrophotometer, Zenyth 200; Anthos, Austria). Results were reported as mM (18).

### Statistical analysis

Data were analyzed by two independent researchers, one was blind to treatment. All data are reported as means±SE of the values of 6 animals. The results were analyzed using two-way ANOVA (factors: lesion and treatment) followed by Tukey *post hoc* test. Differences were considered statistically significant when P was <0.05. Statistical analyses were carried out with the software Statistica 7.0 (USA).

## Results

**Figure 1.**
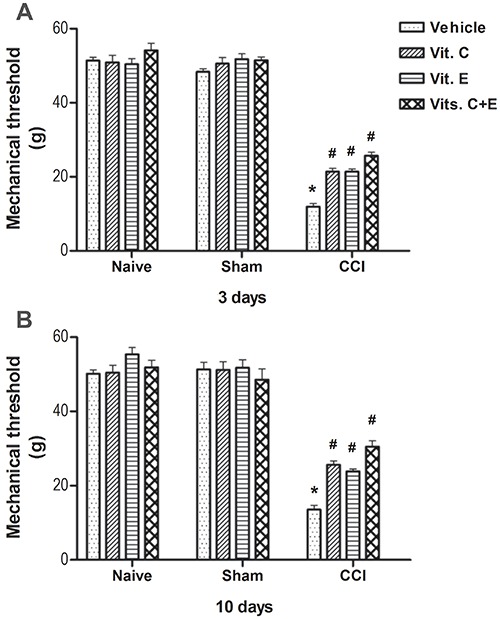
Assessment of mechanical threshold in rats treated with vit. C (30 mg·kg^-1^·day^-1^), vit. E (15 mg·kg^-1^·day^-1^), a combination of these vitamins (vits. C+E) in the same doses, or the vehicle alone (saline containing 1% Tween 80) administered intraperitoneally for 3 (*A*) and 10 (*B*) days after chronic constriction injury (CCI). Data are reported as means±SE (n=6/group). *P<0.05 compared to naive and sham rats and vitamin-treated CCI rats over the same experimental period. ^#^P<0.05 compared to naive and sham rats and vehicle-treated CCI rats over the same experimental period (two-way ANOVA followed by Tukey *post hoc* test).

The mechanical threshold did not change significantly in the naive and sham groups. After CCI, all rats exhibited a decrease in mechanical threshold, which was prevented by vitamins treatment, as showed in our previous study (4). At 3 days after CCI, the mechanical threshold decreased 77% in vehicle-treated CCI rats compared to naive and sham rats. The reductions were of 58, 57, and 52% in CCI rats that received vit. C, vit. E and vits. C+E, respectively, compared to naive and sham rats (Figure 1A). Comparing vehicle and vitamin-treated CCI rats, vits. C+E-treated rats showed an improvement of 116% in the mechanical threshold, whereas the percentages were 77% in vit. C and vit. E-treated CCI rats. At day 10, the mechanical threshold decreased 74% in vehicle-treated CCI rats compared to naive and sham rats. The reductions were of 50, 58, and 42% in CCI rats that received vit. C, vit. E, and vits. C+E, respectively, compared to naive and sham rats (Figure 1B). Comparing vehicle and vitamin-treated CCI rats, vits. C+E-treated rats showed an improvement of 135% in the mechanical threshold, whereas the percentages were 92 and 77% in vit. C- and vit. E-treated CCI rats, respectively.

### Antioxidant parameters

**Figure 2.**
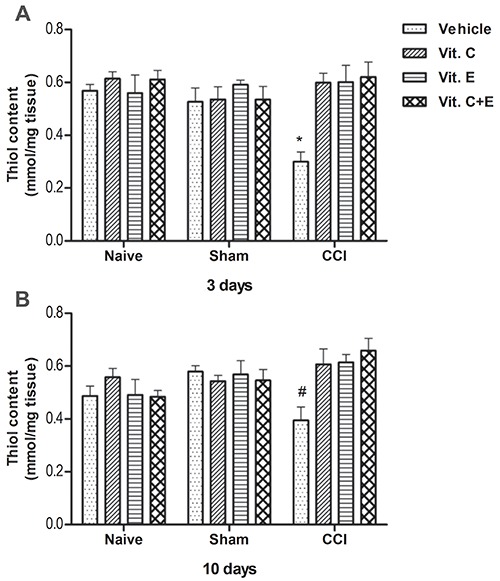
Total thiol content in the spinal cord of rats treated with vit. C (30 mg·kg^-1^·day^-1^), vit. E (15 mg·kg^-1^·day^-1^), a combination of these vitamins (vits. C+E) in the same doses, or the vehicle alone (saline containing 1% Tween 80) administered intraperitoneally for 3 (*A*) and 10 (*B*) days after chronic constriction injury (CCI). Data are reported as means±SE (n=6/group). *P<0.05 compared to naive and sham rats and vitamin-treated CCI rats over the same experimental period. ^#^P<0.05 compared to vitamin-treated CCI rats over the same experimental period (two-way ANOVA followed by Tukey *post hoc* test).

After CCI, the vehicle-treated rats showed significant decreases in the total thiol content at days 3 and 10. At day 3, the reduction was 52.5% compared to naive rats, but it was 43% (P<0.05) compared to sham rats. In vitamin-treated CCI rats, the total thiol content increased 99%, 100% and 106% (P<0.001) in vit. C, vit. E, and vits. C+E CCI rats, respectively, compared to vehicle-treated CCI rats for 3 days (Figure 2A). At day 10, the total thiol content did not show significant change in spinal cord of vehicle-treated CCI rats compared to naive and sham rats, but it was decreased by around 20 and 33% in spinal cord of vehicle-treated CCI rats compared to naive and sham rats, respectively (Figure 2B). In vitamin-treated CCI rats, the total thiol content was similar to that found in naive and sham rats, but it showed significant increase compared to vehicle-treated CCI rats of the same experimental group. The increase was 55.5% (P<0.05), 53.5% (P<0.001) and 66% (P<0.001) in vit. C, vit. E, and vits. C+E-treated CCI rats, respectively.

**Figure 3.**
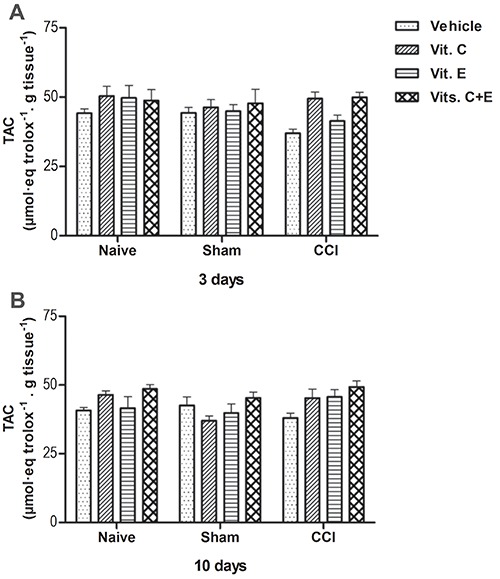
Total antioxidant capacity (TAC) in the spinal cord of rats treated with vit. C (30 mg·kg^-1^·day^-1^), vit. E (15 mg·kg^-1^·day^-1^), a combination of these vitamins (vits. C+E) in the same doses, or the vehicle alone (saline containing 1% Tween 80) administered intraperitoneally for 3 (*A*) and 10 (*B*) days after chronic constriction injury (CCI). Data are reported as means±SE (n=6/group). No statistical difference between groups was found (two-way ANOVA followed by Tukey *post hoc* test).

TAC showed no significant changes in the spinal cord of the vitamins and vehicle-treated CCI rats (Figure 3A and B). Total thiol content and TAC did not change significantly in the naive and sham rats.

### Pro-oxidant parameters

**Figure 4.**
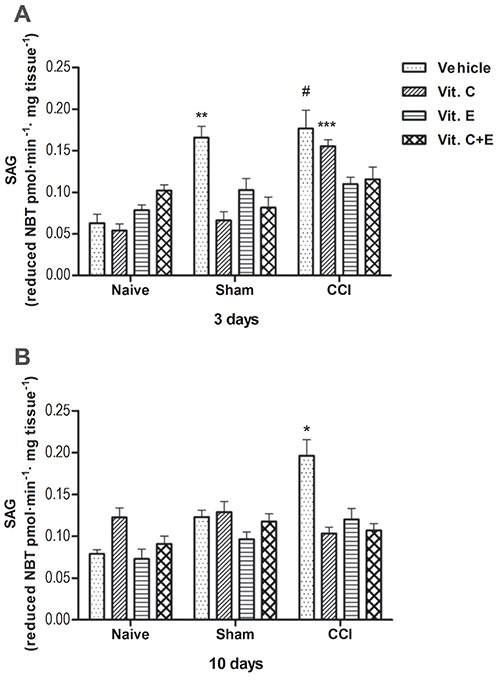
Superoxide anion generation (SAG) in the spinal cord of rats treated with vit. C (30 mg·kg^-1^·day^-1^), vit. E (15 mg·kg^-1^·day^-1^), a combination of these vitamins (vits. C+E) in the same doses, or the vehicle alone (saline containing 1% Tween 80) administered intraperitoneally for 3 (*A*) and 10 (*B*) days after chronic constriction injury (CCI). Data are reported as means±SE (n=6/group). *P<0.05 compared to naive and sham rats and vitamin-treated CCI rats over the same experimental period. **P<0.05 compared to naive rats and vitamin-treated sham rats over the same experimental period. ***P<0.05 compared to naive rats and vit. C and vits. C+E-treated sham rats over the same experimental period. ^#^P<0.05 compared to naive rats, vitamin-treated sham rats, and vit. E and vits. C+E-treated CCI rats over the same experimental period (two-way ANOVA followed by Tukey *post hoc* test).

The level of SAG increased significantly in the lumbosacral spinal cord of vehicle-treated CCI rats. The increase was 157% compared to naive rats, and was found at days 3 and 10 (P<0.001; Figure 4A and B). In vitamin-treated CCI rats, an increase was found only in vit. C-treated CCI rats for 3 days (P<0.001). Rats that received vit. E and vits. C+E for 3 days showed SAG levels that were similar to those found in naive rats. The vit. E and vits. C+E-treated CCI rats showed significant decrease in SAG levels compared to vehicle-treated CCI rats of the same experimental group (P<0.05). At day 10 post-CCI, all rats that received vitamins showed SAG levels that were similar to those found in naive rats. The SAG levels decreased 53, 44, and 55% in CCI rats that received vit. C, vit. E, or a combination of vits. C+E, respectively, compared to vehicle-treated CCI rats of the same experimental group (P<0.001). In sham rats, the level of SAG increased only in rats that received vehicle for 3 days compared to naive rats and vitamin-treated sham rats (P<0.05). No significant change in the levels of SAG was found in naive rats.

**Figure 5.**
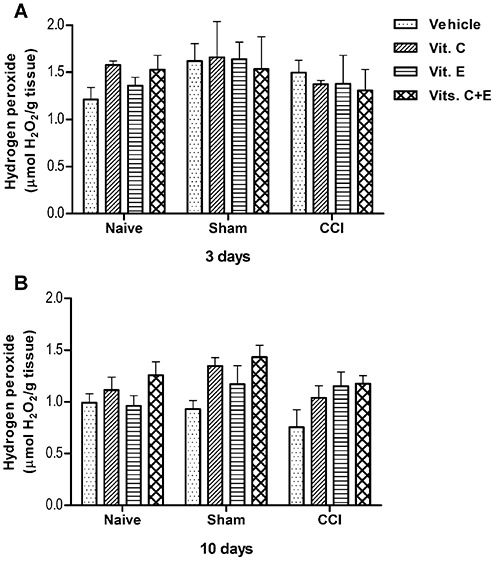
Hydrogen peroxide levels in the spinal cord of rats treated with vit. C (30 mg·kg^-1^·day^-1^), vit. E (15 mg·kg^-1^·day^-1^), a combination of these vitamins (vits. C+E) in the same doses, or the vehicle alone (saline containing 1% Tween 80) administered intraperitoneally for 3 (*A*) and 10 (*B*) days after chronic constriction injury (CCI). Data are reported as means±SE (n=6/group). No statistical difference between groups was found (two-way ANOVA followed by Tukey *post hoc* test).

**Figure 6.**
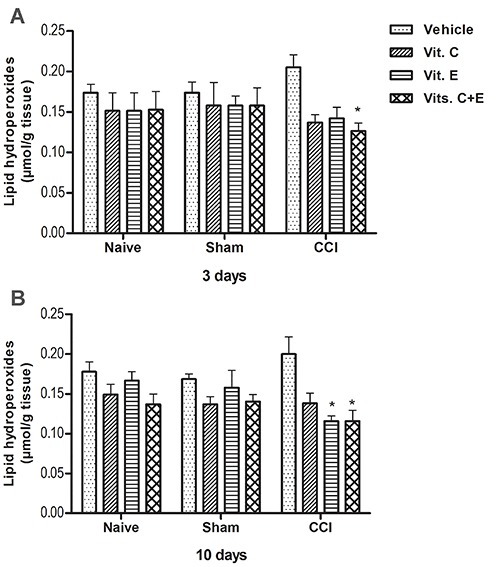
Lipid hydroperoxides levels in the spinal cord of rats treated with vit. C (30 mg·kg^-1^·day^-1^), vit. E (15 mg·kg^-1^·day^-1^), a combination of these vitamins (vits. C+E) in the same doses, or the vehicle alone (saline containing 1% Tween 80) administered intraperitoneally for 3 (*A*) and 10 (*B*) days after chronic constriction injury (CCI). Data are reported as means±SE (n=6/group). *P<0.05 compared to vehicle-treated CCI rats over the same experimental period (two-way ANOVA followed by Tukey *post hoc* test).

H_2_O_2_ levels showed no significant changes at the times assessed (Figure 5A and B). Lipid hydroperoxides showed no significant change in the spinal cord of vehicle-treated CCI rats (Figure 6A and B). However, lipid hydroperoxides increased by around 25% in these rats at days 3 and 10 compared to naive rats. This increase was not observed in CCI rats that received vitamins. At day 3, while lipid hydroperoxides did not show significant change in spinal cord of vit. C and vit. E-treated CCI rats, the levels significantly reduced in vits. C+E-treated CCI rats compared to vehicle-treated CCI rats of the same experimental group (43%, P<0.05). At day 10, the lipid hydroperoxides significantly decreased (43%) in spinal cord of vit. E and vits. C+E-treated CCI rats (P=0.0021) compared to vehicle-treated CCI rats of the same experimental group. Despite not significant, the lipid hydroperoxides decreased 33% in spinal cord of vit. C-treated CCI rats. No significant change was found in sham and naive rats.

**Figure 7.**
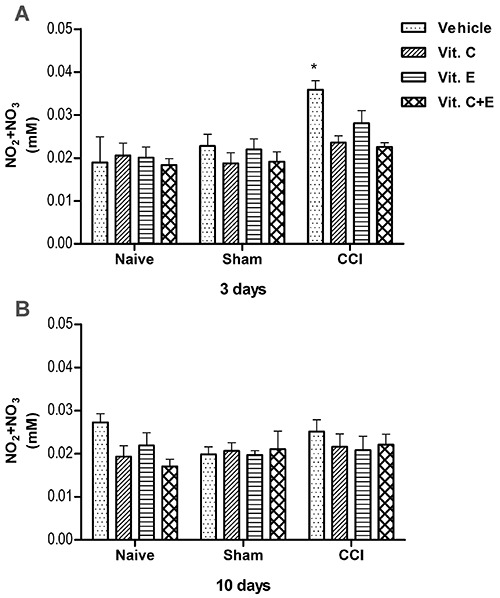
Nitric oxide metabolites (NO_2_+NO_3_) in the spinal cord of rats treated with vit. C (30 mg·kg^-1^·day^-1^), vit. E (15 mg·kg^-1^·day^-1^), a combination of these vitamins (vits. C+E) in the same doses, or the vehicle alone (saline containing 1% Tween 80) administered intraperitoneally for 3 (*A*) and 10 (*B*) days after chronic constriction injury (CCI). Data are reported as means±SE (n=6/group). *P<0.05 compared to naive and sham rats and vit. C and vits. C+E-treated CCI rats over the same experimental period (two-way ANOVA followed by Tukey *post hoc* test).

The NO metabolites increased 90% (P<0.05) in the spinal cord of CCI rats that received the vehicle for 3 days, compared to naive and sham rats (Figure 7A). At day 10, this increase was not observed (Figure 7B). In the spinal cord of the vitamin-treated CCI rats, the level of NO metabolites was similar to that found in naive rats at both times. No significant change was found in sham and naive rats.

## Discussion

The first point to be clarified in our study is the decision to use the rat as an experimental model, even though rats can synthesize their own ascorbic acid (19). Despite this characteristic, rats are frequently used as an experimental model to study the effects of vit. C treatment on nerve tissue (4,20). Rats are also the most frequently used laboratory animals for experimental models of peripheral neuropathic pain (6). Therefore, the rat is a valid model to study the effect of treatment with vit. C on neuropathic pain, a treatment that still has many unanswered questions.

Our study focused on effects of vit. C and vit. E, given alone or in combination, on oxidative-stress parameters in the spinal cord of CCI rats, because of the emerging role of ROS in pain mechanisms (2,21). While the vehicle-treated CCI rats showed a decrease in total thiol content, this reduction was not found in the vitamin-treated CCI rats. Decreased glutathione, the most abundant thiol in mammals, was also observed by other investigators in the spinal cord of CCI rats (22). Total thiols constitute a group of molecules that act as cofactors in some enzymatic systems, and they can directly neutralize radicals (23). The observed decrease in total thiols could be due to their depletion as a result of the increased production of ROS in the spinal cord of vehicle-treated CCI rats. ROS can mediate the occurrence and maintenance of neuropathic pain (2,7). The lack of a decrease in total thiol content of the vitamin-treated CCI rats may be related to the antioxidant role of the vitamins. Bütün et al. (24) demonstrated that vit. E had a protective effect on the glyceryl trinitrate-induced brain injury by inhibiting free radical production, regulating the calcium-dependent process, and supporting the antioxidant redox system. Vit. C directly acts to scavenge oxygen- or nitrogen-based radical species generated during cellular metabolism (25). In addition, vit. C transforms vit. E to its active form (24,26). Therefore, it appears valid to suggest that the antioxidant activity of the vitamins prevented the decrease in total thiol content of the spinal cord.

Interestingly, TAC did not change significantly in the spinal cord of the vitamin-treated rats. This result may be related to a tight control of the vitamins to ensure a pro-oxidant state in these animals. According to Poljsak et al. (27), excessive ROS formation needs to be corrected only to prevent the accumulation of oxidative damage, and a slight pro-oxidative balance is necessary for optimal cell-signaling processes. At low levels, free radicals regulate the activities of different transcription factors and protein-signaling cascades (28). However, TAC did not change in the spinal cord of the vehicle-treated CCI rats, which showed a decrease in total thiols. It has been demonstrated that TAC represents the enzymatic and non-enzymatic antioxidant compounds in the body such as superoxide dismutase, catalase, glutathione peroxidase and glutathione (29). CCI increases catalase activity in the spinal cord 3 and 10 days after surgery (30). Catalase is an antioxidant enzyme, located in peroxisomes, which catalyzes the breakdown of H_2_O_2_ to H_2_O and O_2_ (2). The increase in catalase activity may be related to a lack of significant change in TAC in the spinal cord of the vehicle-treated CCI rats.

In our study, administration of vitamins reduced the levels of SAG in the spinal cord of the CCI rats, which increased in the vehicle-treated CCI rats. It has been demonstrated that vit. C and vit. E decrease the superoxide anion concentration and the activation of the NADPH oxidase, the major source of superoxide anions (31). Thus, these effects of vitamins may be contributing to decreased level of SAG in the spinal cord of the vitamin-treated CCI rats. However, while the vit. E and vits. C+E-treated CCI rats showed reductions in these levels at days 3 and 10, the vit. C-treated CCI rats showed a decrease only at day 10. This difference may be related to pro-oxidant properties of vit. C. Ascorbate readily undergoes pH-dependent autoxidation, producing H_2_O_2_ (32). This appears to occur because parenteral administration bypasses the tight control of ascorbate, which is restored as the kidneys excrete ascorbate when concentrations are higher than those corresponding to V_max_ of the reabsorptive transporters (32). According to these authors, when the tight control of ascorbate is bypassed, H_2_O_2_ forms in the extracellular space, and as tight control is restored, H_2_O_2_ formation ceases. Our results suggest that tight control of vit. C was restored at day 10, but not at day 3. The lack of change in H_2_O_2_ levels may be related to activity of antioxidant enzymes such as superoxide dismutase and catalase. Superoxide dismutase converts superoxide to H_2_O_2_ (2), which is breakdown by catalase, as discussed above. Furthermore, it has been demonstrated that vit. C increases superoxide dismutase activity (31). Although not assessed in our study, it appears important to determine the effect of vitamins on activities of these enzymes in spinal cord of CCI rats.

The increase in SAG levels in vehicle-treated CCI rats at 3 and 10 days may be related to neuropathic pain. Significant evidence links the superoxide anion to pain of several etiologies, including neuropathic pain (2,33). Recently we demonstrated that CCI rats that received the same vehicle used in the present study did not exhibit recovery in the mechanical threshold 3 and 10 days after surgery. However, vitamin-treated CCI rats showed antinociception in these times (4). In addition, vit. C ameliorates pain in humans (34). The antinociceptive effect may be related to antioxidant actions of the vitamins discussed above. It has been suggested that restoring nitrosative balance in peripheral and central nervous system is a possible therapeutic approach for ameliorating neuropathology (2). This suggestion does not exclude other functions of vitamins that could be contributing to antinociception. Vit. E regulates the calcium-dependent process (24), and vit. C has a function as cofactor for biosynthesis of amidated opioid peptides and a family of biosynthetic and regulatory metallo-enzymes (34). All these mechanisms may be involved in the vitamin-induced antinociception, as well as the antioxidant actions of the vitamins.

Our study also showed that vitamins, given alone or in combination, prevented the increase in NO metabolites in the spinal cord, which were increased in vehicle-treated CCI rats after 3 days. The increase in NO metabolites at day 3 but not at day 10 was also found by other authors (3). The lack of this increase in spinal cord of vitamin-treated CCI rats may be related to the effect of vit. C and vit. E on calcium channels. NO formation is induced by nitric-oxide synthase activation in a calcium/calmodulin-dependent manner following stimulation of calcium-permeable N-methyl-d-aspartate (NMDA) receptors (35). Vit. E inhibits the activation of the oxidative stress-induced melastatin-like transient receptor potential 2 (TRPM2) channel, which is an oxidative redox-sensitive calcium-permeable cation channel (36). Vit. C inhibits the C_av_3.2 isoform of T-type calcium channels involved in neuropathic pain (37). These actions of vit. C and vit. E may help to prevent the increase in the NO metabolites in the spinal cord of the vitamin-treated CCI rats. The increase in NO metabolites in the spinal cord of the vehicle-CCI rats may be related to the role of NO in neuropathic pain. NO is an important neurotransmitter involved in the nociceptive process, which is increased in rats with neuropathic pain (38). The reduction in total thiol content may also be related to the increase in NO. Glutathione plays an important role in NO availability. Glutathione reacts with peroxynitrite from S-nitrosothiols, which subsequently release NO over a prolonged period to extend the half-life of NO (39). The reaction of glutathione with peroxynitrite may also have contributed to the decrease in total thiols.

The effects of the vitamins discussed above may have contributed to the decrease in lipid hydroperoxides in the spinal cord of the vitamin-treated CCI rats compared to the vehicle-treated CCI rats. The small increase in the lipid hydroperoxides in the spinal cord of vehicle-treated CCI rats may be related to increased levels of SAG and NO found in these rats. Since excessive ROS formation needs to be corrected only to prevent the accumulation of oxidative damage (27,28), this may explain the small increase in lipid hydroperoxides. However, reactive aldehyde production occurs by lipid peroxidation of mitochondrial and plasma membranes from ROS, inducing pain (40). Thus, the increase in the lipid hydroperoxides in the vehicle-treated CCI rats may be indicating increased levels of reactive aldehyde production. The lack of an increase in lipid hydroperoxides of vitamin-treated CCI rats may be related to the effects of the vitamins on reactive aldehyde production. However, this suggestion needs further study.

The lack of significant change in H_2_O_2_ may be also related to the effects of these vitamins as antioxidants and/or inhibitors of calcium channels, as discussed above. The lack of change in H_2_O_2_ of vehicle-treated CCI rats may be related to an increase in catalase activity in the spinal cord of rats with CCI (30).

The increase in the levels of SAG in the spinal cord of vehicle-treated sham rats may be due to the procedures involving manipulation of deep tissues, such as muscles and adjacent connective tissue, which induce pain (3,21). Since administration of vitamins C and E, given alone or in combination, induced an antinociceptive effect in these animals, this result may be related to the antioxidant actions of the vitamins discussed above. In addition, the effect of the vitamins in sham rats reinforces the vitamin-induced antinociception in pain conditions.

In conclusion, this study provided evidence that administration of vitamins C and E, given alone or in combination, prevented changes in pro-oxidant and antioxidant markers in the spinal cord of CCI rats. In these rats, CCI induced an increase in SAG and NO metabolites and decreased total thiol content. These changes were not found in the spinal cord of CCI rats that received vitamins. Since a previous study showed that the same treatment protocol induced antinociception in CCI rats (4), the findings of the current study may be related to some role in the antinociceptive effect of these vitamins, because the scavenging of ROS appears to help normalize the spinal cord oxidative status altered by pain.

## References

[1] Colloca L, Ludman T, Bouhassira D, Baron R, Dickenson AH, Yarnitsky D (2017). Neuropathic pain. Nat Rev Dis Primers.

[2] Grace PM, Gaudet AD, Staikopoulos V, Maier SF, Hutchinson MR, Salvemini D (2016). Nitroxidative signaling mechanisms in pathological pain. Trends Neurosci.

[3] Horst A, Kolberg C, Moraes MS, Finamor IA, Belló-Klein A, Pavanato MA (2014). Effect of N-acetylcysteine on the spinal-cord glutathione system and nitric-oxide metabolites in rats with neuropathic pain. Neurosc Lett.

[4] Riffel AP, de Souza JA, Santos M C, Horst A, Scheid T, Kolberg C (2016). Systemic administration of vitamins C and E attenuates nociception induced by chronic constriction injury of the sciatic nerve in rats. Brain Res Bull.

[5] Wawrzyniak A, Górnicka M, Hamułka J, Gajewska M, Drywień M, Pierzynowska J (2013). α-Tocopherol, ascorbic acid and β-carotene protect against oxidative stress but reveal no direct influence on p53 expression in rats subjected to stress. Nutr Res.

[6] Jaggi AS, Jain V, Singh N (2011). Animal models of neuropathic pain. Fundam Clin Pharmacol.

[7] Kim HY, Lee I, Chun SW, Kim HK (2015). Reactive oxygen species donors increase the responsiveness of dorsal horn neurons and induce mechanical hyperalgesia in rats. Neural Plast 2015.

[8] Bruno RS, Leonard SW, Atkinson J, Montine TJ, Ramakrishnan R, Bray TM (2006). Faster plasma vitamin E disappearance in smokers is normalized by vitamin C supplementation. Free Radic Bio Med.

[9] Halliwell B (2006). Oxidative stress and neurodegeneration: where are we now?. J Neurochem.

[10] Kontush A, Mann U, Arlt S, Ujeyl A, Lührs C, Müller-Thomsen T (2001). Influence of vitamin E and C supplementation on lipoprotein oxidation in patients with Alzheimer's disease. Free Radic Biol Med.

[11] Lu R, Kallenborn-Gerhardt W, Geisslinger G, Schmidtko A (2011). Additive antinociceptive effects of a combination of vitamin c and vitamin e after peripheral nerve injury. Plos One.

[12] Bennett GJ, Xie YK (1988). A peripheral mononeuropathy in rat that produces disorders of pain sensation like those seen in man. Pain.

[13] Aksenov MY, Markesbery WR (2001). Changes in thiol content and expression of glutathione redox system genes in the hippocampus and cerebellum in Alzheimer's disease. Neurosci Lett.

[14] Erel O (2004). A novel automated direct measurement method for total antioxidant capacity using a new generation, more stable ABTS radical cation. Clin Biochem.

[15] Wang HD, Pagano PJ, Du Y, Cayatte AJ, Quinn MT, Brecher P (1998). Superoxide anion from the adventitia of the rat thoracic aorta inactivates nitric oxide. Circ Res.

[16] Pick E, Keisari Y (1980). A simple colorimetric method for the measurement of hydrogen peroxide produced by cells in culture. J Immunol Methods.

[17] Jiang ZY, Woollard ACS, Wolff SP (1991). Lipid hydroperoxide measurement by oxidation of Fe^2+^ in the presence of xylenol orange. Comparison with the TBA assay and an iodometric method. Lipids.

[18] Granger DL, Taintor RR, Boockvar KS, Hibbs JB (1996). Measurement of nitrate and nitrite in biological samples using nitrate reductase and Griess reaction. Methods Enzymol.

[19] Rice ME (2000). Ascorbate regulation and its neuroprotective role in the brain. Trends Neurosci.

[20] Lee JY, Choi HY, Yune TY (2016). Fluoxetine and vitamin C synergistically inhibits blood-spinal cord barrier disruption and improves functional recovery after spinal cord injury. Neuropharmacology.

[21] Scheid T, Bosco LD, Guedes RP, Pavanato MA, Belló-Klein A, Partata WA (2013). Sciatic nerve transection modulates oxidative parameters in spinal and supraspinal regions. Neurochem Res.

[22] Bhat RA, Lingaraju MC, Pathak NN, Kalra J, Kumar D, Tandan SK (2016). Effect of ursolic acid in attenuating chronic constriction injury-induced neuropathic pain in rats. Fundam Clin Pharmacol.

[23] Sies H (1999). Glutathione and its role in cellular functions. Free Radic Biol Med.

[24] Bütün A, Nazıroğlu M, Demirci S, Çelik Ö, Uğuz AC (2015). Riboflavin and vitamin E increase brain calcium and antioxidants, and microsomal calcium-ATP-ase values in rat headache models induced by glyceryl trinitrate. J Membr Biol.

[25] Harrison FE, May JM (2009). Vitamin C function in the brain: Vital role of the ascorbate transporter (SVCT2). Free Radic Biol Med.

[26] Daiber A, Daub S, Bachschmid M, Schildknecht S, Oelze M, Steven S (2013). Protein tyrosine nitration and thiol oxidation by peroxynitrite-strategies to prevent these oxidative modifications. Int J Mol Sci.

[27] Poljsak B, Suput D, Milisav I (2013). Achieving the balance between ROS and antioxidants: when use the synthetic antioxidants. Oxid Med Cell Longev.

[28] Kaminskyy VO, Zhivotovsky B (2014). Free radicals in cross talk between autophagy and apoptosis. Antioxid Redox Signal.

[29] Şahin A, Erten S, Altunoğlu A, Işikoğlu S, Neşelioğlu S, Ergin M (2014). Comparison of serum oxidant and antioxidant parameters in familial Mediterranean fever patients with attack free period. Acta Reumatol Port.

[30] Goecks CSB, Horst A, Moraes MS, Scheid T, Kolberg C, Bello-Klein A (2012). Assessment of oxidative parameters in rat spinal cord after chronic constriction of the sciatic nerve. Neurochem Res.

[31] Chen X, Touyz RM, Park JB, Schiffrin EL (2001). Antioxidant effects of vitamins C and E are associated with altered activation of vascular NADPH oxidase and superoxide dismutase in stroke-prone SHR. Hypertension.

[32] Chen Q, Espey MG, Sun AY, Lee JH, Krishna MC, Shacter E (2007). Ascorbate in pharmacologic concentrations selectively generates ascorbate radical and hydrogen peroxide in extracellular fluid *in vivo*. Proc Natl Acad Sci USA.

[33] Little JW, Doyle T, Salvemini D (2010). Reactive nitroxidative species and nociceptive processing: determining the roles for nitric oxide, superoxide, and peroxynitrite in pain. Amino Acids.

[34] Carr AC, McCall C (2017). The role of vitamin C on the treatment of pain: new insights. J Transl Med.

[35] Mukherjee P, Cinelli MA, Kang S, Silverman RB (2014). Development of nitric oxide synthase inhibitors for neurodegeneration and neuropathic pain. Chem Soc Rev.

[36] Nazıroğlu M, Özgül C (2013). Vitamin E modulates oxidative stress and protein kinase C activator (PMA)-induced TRPM2 channel gate in dorsal root ganglion of rats. J Bioenerg Biomembr.

[37] Nelson MT, Joksovic PM, Su P, Kang HW, Van Deusen A, Baumgart JP (2007). Molecular mechanisms of subtype-specific inhibition of neuronal T-type calcium channels by ascorbate. J Neurosci.

[38] Zhou Z, Liang Y, Deng F, Cheng Y, Sun J, Guo L (2015). Phosphorylated neuronal nitric oxide synthase in neuropathic pain in rats. Int J Clin Exp Pathol.

[39] Robaczewska J, Kedziora-kornatowska K, Kozakiewicz M, Zary-Sikorska E, Pawluk H, Pawliszak W (2016). Role of glutathione metabolism and glutathione-related Antioxidant defense systems in hypertension. J Physiol Pharmacol.

[40] Zambelli VO, Gross ER, Chen C-H, Gutierrez VP, Cury Y, Mochly-Rosen D (2014). Aldehyde dehydrogenase-2 regulates nociception in rodent models of acute inflammatory pain. Sci Transl Med.

